# Surface-Enhanced Raman Spectroscopy (SERS)-Based Sensors for Deoxyribonucleic Acid (DNA) Detection

**DOI:** 10.3390/molecules29143338

**Published:** 2024-07-16

**Authors:** Shireen Zangana, Miklós Veres, Attila Bonyár

**Affiliations:** 1 Department of Electronics Technology, Faculty of Electrical Engineering and Informatics, Budapest University of Technology and Economics, 1111 Budapest, Hungary; shireen.zangana@edu.bme.hu; 2 HUN-REN Wigner Research Centre for Physics, 1525 Budapest, Hungary; veres.miklos@wigner.hun-ren.hu

**Keywords:** Raman spectroscopy, surface-enhanced Raman spectroscopy, SERS, DNA detection, enhancement factor

## Abstract

Surface-enhanced Raman spectroscopy (SERS) has emerged as a powerful technique for the detection and analysis of biomolecules due to its high sensitivity and selectivity. In recent years, SERS-based sensors have received significant attention for the detection of deoxyribonucleic acid (DNA) molecules, offering promising applications in fields such as medical diagnostics, forensic analysis, and environmental monitoring. This paper provides a concise overview of the principles, advancements, and potential of SERS-based sensors for DNA detection. First, the fundamental principles of SERS are introduced, highlighting its ability to enhance the Raman scattering signal by several orders of magnitude through the interaction between target molecules with metallic nanostructures. Then, the fabrication technologies of SERS substrates tailored for DNA detection are reviewed. The performances of SERS substrates previously reported for DNA detection are compared and analyzed in terms of the limit of detection (LOD) and enhancement factor (EF) in detail, with respect to the technical parameters of Raman spectroscopy (e.g., laser wavelength and power). Additionally, strategies for functionalizing the sensor surfaces with DNA-specific capture probes or aptamers are outlined. The collected data can be of help in selecting and optimizing the most suitable fabrication technology considering nucleotide sensing applications with Raman spectroscopy.

## 1. Introduction

Surface-enhanced Raman spectroscopy (SERS) is a potent analytical technique that shows great promise in biochemistry, materials research, and environmental monitoring. It provides exceptional sensitivity and specificity for detecting and characterizing molecular species at the nanoscale by utilizing the concepts of Raman scattering and plasmonics.

In recent years, SERS has received substantial attention in nucleic acid analysis, particularly for detecting deoxyribonucleic acid (DNA). Detecting DNA is crucial in various fields, such as clinical diagnostics, forensic investigations, agricultural genomics, and environmental monitoring.

Formerly, DNA detection has depended on laborious and time-consuming techniques like gel electrophoresis, polymerase chain reaction (PCR), and fluorescence-based tests. Although these methods have advantages for revealing genetic information, they have drawbacks such as low sensitivity, a restricted ability to analyze many targets simultaneously, and vulnerability to contamination. Furthermore, several traditional DNA detection techniques necessitate sophisticated equipment and trained staff, limiting their broad implementation and accessibility, especially in resource-constrained environments.

In contrast, SERS is a promising method for detecting DNA using the distinctive optical characteristics of plasmonic nanostructures to amplify Raman signals from molecular substances. SERS-based sensors demonstrate exceptional sensitivity, are able to detect low amounts of specific target molecules with remarkable precision and require minimal sample preparation. SERS possesses multiplexing capabilities, allowing for the simultaneous detection of several DNA sequences in a single examination, which enhances workflow efficiency and increases throughput.

### 1.1. Raman Spectroscopy and SERS

Raman scattering is a phenomenon of inelastic light scattering, where the energy of an incoming photon alters because of the interaction with the distinctive molecular vibrations. During this process, the scattered photon can either possess lower energy (Stokes Raman scattering) or higher energy (anti-Stokes Raman scattering), depending on whether the vibration is excited or annihilated. Importantly, the energy difference between the incident and scattered photons always corresponds to the energy associated with the particular vibration being involved.

Being sensitive to molecular vibrations affected by composition and topology, Raman spectroscopy provides a fingerprint spectrum of a molecule, consisting of peaks related to its possible vibrations, the excitation of which is allowed by the Raman process (selection rules) and can be used to characterize its bonding configuration. Raman spectra are recorded by exciting the sample with a monochromatic light source and recording the scattered light spectrum at wavelengths different from that of the excitation source. In general, the Raman process involves the excitation of an electron by the incident photon to a virtual energy state (where it excites/annihilates the vibration).

The Raman effect is limited by its sensitive and relatively weak nature. Raman scattering’s low occurrence probability is due to its intrinsic weakness compared with other processes like Rayleigh scattering. In general, the cross-section of the Raman scattering processes (~10^−31^ cm^2^/sr) is cca. 4 orders of magnitude smaller than that of Rayleigh scattering (10^−27^ cm^2^/sr), while this value, for the infrared absorption (~10^−20^ cm^2^/sr) and fluorescence emission (~10^−16^ cm^2^/sr), is much higher [1]. In terms of concentration, the detection limit of Raman spectroscopy is at the ppb-ppm or nanomolar level, depending on the analyte [2]. Lower concentrations of analytes or materials are hard to detect because of the very low intensity of the Raman signal, which is comparable to or below the noise level. 

However, if the excitation photon energy is equal to the energy of an existing electronic transition of the material, resonant Raman scattering occurs, which is two to three orders of magnitude stronger than the non-resonant Raman effect [3].

Besides the resonant Raman scattering, surface-enhanced Raman scattering can also amplify the Raman signal, in which the presence of some nanostructured materials enormously enhances the Raman scattering of the molecules in their vicinity. These are materials with plasmonic properties, with a resonance wavelength in the Raman excitation and/or inelastically scattered light’s wavelength region. SERS has great potential in improving Raman-based sensing. Its proven single-molecule detection ability drives the research to develop SERS enhancement platforms for detecting molecules on a sub-ppb or even lower level [4]. Due to water’s relatively weak SERS spectrum [5], SERS is highly efficient in studying biological samples and detecting biomolecules even at low concentrations [6]. SERS is also available with dielectrics and semiconductors but with a much smaller enhancement than with metal-based plasmonic structures [7].

SERS is a phenomenon in which the localized surface plasmon resonance (LSPR) of metallic nanoparticles or other nanostructures significantly amplifies the electromagnetic field involved in Raman scattering from molecules adsorbed onto these metallic surfaces. This amplification leads to enhanced sensitivity and an improved signal-to-noise ratio.

The observation of the SERS effect dates back to 1974 when Fleischmann and his research team unexpectedly discovered enhanced Raman peaks for pyridine adsorbed onto a roughened silver electrode. It was subsequently observed that arranging molecules on nanoparticle surfaces or metal surfaces with nanoscale roughness leads to a disproportionate increase in their Raman scattering intensity, regardless of the molecule concentration. Further investigations revealed that the SERS effect diminishes rapidly with distance from the metal surface, and the properties of the metal itself play a critical role in this phenomenon. Specifically, noble metal surfaces with rough textures, particularly gold and silver, have the capability to generate LSPRs when excited by light of a suitable photon energy. These resulting plasmon fields can significantly enhance the intensity of the Raman signals emitted by molecules in close proximity to the metal surface [8]. 

Various theories have been put forth to elucidate the SERS effect, and currently, it is widely acknowledged that two mechanisms contribute to this phenomenon: electromagnetic (EM) enhancement and chemical enhancement (CE). The EM mechanism postulates that the enhancement arises from the resonant excitation of the metal’s oscillating electrons (plasmons) by the electromagnetic field of the incident and/or scattered light [9]. During this resonant coupling, the metal acts as an amplifying nanoantenna that could increase the electromagnetic field of the excitation/scattered light in its close vicinity typically by 10–100 times, resulting in a Raman signal with an intensity of five to nine orders of magnitude higher intensity. The EM SERS enhancement strongly depends on the properties of the plasmonic metal surface, including its material, size, shape, and, in the case of SERS substrates, surface roughness [10].

Since the Raman effect involves excitation and scattered photons of two different energies, the EM enhancement has two distinct components. One is the near-field enhancement, in which the plasmons induce robust spatial localization and amplify the excitation light in small regions called hotspots. Around these regions, the molecules experience an electromagnetic field much stronger than the standard excitation field, resulting in high levels of Raman scattering. The other is the so-called re-radiation enhancement related to the amplification of the Raman scattered light emitted by the Raman scattering molecule as a dipole and affected by the interface of the plasmonic surface. While the emission pattern of a dipole is symmetric in a vacuum or homogenous medium, it could be scattered and partly backscattered to the dipole by nearby interfaces. This backscattered radiation (or self-reaction field) could influence the radiation emitted by the dipole and could amplify the Raman scattering [10].

The chemical enhancement mechanism, proposed by Albrecht and Creighton [11], is based on a charge transfer effect between the molecule and the metal that is related to the formation of new electronic level(s) when the molecule involved in Raman scattering is attached to the metal surface. In many cases, this chemical enhancement is accompanied by the appearance of new bands in the SERS spectrum. CE could contribute to SERS in two different ways [11]. In resonant charge transfer, the interaction of the molecule and the metal leads to the formation of a metal–molecule charge transfer state that is involved in resonant Raman scattering, giving a remarkably higher Raman signal. During the non-resonant CE, there is no resonance of the new electronic states. However, the interaction with the metal surface induces changes in the geometry and electronic structure of the molecule, altering the probability of specific Raman transitions and resulting in enhanced Raman intensities and a slight modification of the Raman peak positions.

In spite of its potential for sensing with high sensitivity and specificity, several bottlenecks limit the broad practical application of SERS. The plasmon resonances involved in the enhancement are typically wavelength-dependent. Therefore, while the SERS and normal Raman spectrum of most molecules are very similar, the amplification shows wavelength dependence. In addition, the preferred orientation of the molecule on the surface of the SERS substrate could change the relative intensities of the peaks in the SERS spectrum, while new bands can also be present (or known ones missing) due to the charge transfer state formation described above. The polarization dependence of the spectrum can also be affected by SERS [12]. These factors encumber the analysis and interpretation of the SERS spectra.

Photobleaching is involved in the SERS measurements of some analytes. In addition to the decrease in the peak intensities, this effect could also be accompanied by photoinduced chemical reactions and the formation of photoproducts, the Raman bands of which could interfere with the Raman spectrum of the analyte. Photobleaching, together with the dynamic changes in the SERS substrate configuration (hotspot geometry; motion of the colloidal particles), could also contribute to the SERS signal fluctuations (blinking effect), impeding quantitative analysis. The presence of photobleaching and intensity fluctuations has to be analyzed on a case-by-case basis, and their impact can be reduced by using internal standards during the measurements [13].

The reproducibility and uniformity of the SERS substrates is another, perhaps the most relevant, issue impeding the wide spread of practical SERS applications. The performance of even the simplest SERS substrate, made of nanoparticles, freely suspended in a homogeneous medium, is remarkably affected by aggregation (sometimes initiated by the analyte). This effect results in randomly organized combinations and geometries with local hotspots, the enhancement of which could be much larger than that of single particles. In addition, these aggregates tend to sediment, making the SERS signal unstable and fluctuate [14].

An uneven distribution and inherent structural instability of the hotspots are also characteristic of nanoparticle/nanostructure SERS arrays prepared on solid substrates. The hotspots may undergo laser-induced melting or show the diffusion of surface atoms, changing the size, shape, and interparticle distance of the surface pattern. The diffusion of surface atoms is caused by their lower coordination number compared with that of interior atoms. Nanorods and particles with sharp angles, like nanostars or nanocubes, are more affected by this effect, which could reshape the nanoparticles into a more stable (spherical) structure [1]. In the case of solid SERS substrates, stable performance over a large area is also affected by the limitations of the fabrication technology. While SERS has proven outstanding sensitivity and specificity, as demonstrated in many applications, its real-world applications require further developments in SERS substrates, the fine-tuning of the measurement conditions, and novel methods for handling the SERS-specific alterations in the Raman spectra of the analytes.

### 1.2. SERS-Based DNA Sensing

Detection of specific DNA sequences is of great importance in modern biomedicine. This serves as a basis for diagnosing genetic diseases, gene mutations, gene expression profiling, or circulating free tumor DNA, and it is also used in bioanalytical chemistry and forensic science [15]. 

As in most SERS applications, the substrate also plays a critical role in DNA sensing. Many groups reviewed distinct aspects of SERS DNA sensing, including the use of gold nanoparticles (AuNPs) for detecting DNA targets [16,17], SERS-based sensors for COVID-19 investigations [18], aptamer-based SERS biosensors [19], DNA sensing for food safety and environmental monitoring [20], and SERS characterization of biomolecules [21,22,23]. Also, others [24] have studied the factors that affect sensitivity and SERS intensity. At the same time, few have examined the difficulties and challenges of producing such sensors [25]. In this work, a comparison is performed between the surveyed articles on DNA detection via SERS.

SERS DNA sensing has two approaches: label-free and label-based. The label-free methods measure the intrinsic Raman signature of the target analyte (DNA) interacting with the SERS substrate. The most important features, the native DNA-related peaks with their assignments, are listed in Table 1. Although label-free methods are simpler, faster, cheaper, and generally favorable in most biosensing applications, in the case of the SERS detection of DNA hybridization, selectivity (distinguishing signals from the probe and target ss-DNA in a quantitative manner) is always an issue. For this reason, label-based techniques have a stable place in the SERS detection of DNA. Such techniques utilize a highly sensitive and distinctive SERS label (or tag) that is attached to either part of the SERS substrate that has a response to the binding of the target DNA or directly to the target. Later, in Table 2, we indicate the type of sensing approach (label-free or labeled) for all surveyed works. The most frequently used Raman labels for DNA detection are also listed in the caption of Table 2. Both label-free and label-based approaches highly depend on the interaction with the SERS substrates; therefore, manipulating and controlling how the SERS substrate interacts with the target analyte is very important [26,27,28,29,30,31,32].

Various methodologies have been developed for SERS-based DNA detection, encompassing direct detection, sandwich assays, and hybridization-based approaches. These methodologies offer versatility and adaptability regarding target detection, sample compatibility, and assay throughput. SERS-based sensors have found successful applications across diverse domains, including clinical diagnostics, environmental monitoring, food safety, and forensic analysis. Recent advancements in nanofabrication techniques, surface functionalization, and signal processing algorithms have further augmented SERS-based DNA detection platforms’ sensitivity, reproducibility, and reliability.

In this work, we will collect the most important factors that affect DNA sensing with SERS and survey previously published solutions based on their performance, with a special focus on nanofabrication technologies. The reports on successful SERS-based DNA detection are summarized in Table 2, where the main parameters of the SERS substrates, the tested DNA samples, and the SERS enhancement factors and limits of detection (LOD) are also provided (depending on their availability in the cited papers).

### 1.3. Factors Affecting DNA Sensing Performance with SERS

As in most SERS applications, the type and properties of the substrate play a critical role in DNA sensing. The choice of SERS substrate for DNA sensing depends on specific application requirements, such as sensitivity, specificity, throughput, and portability. By understanding the performance characteristics of different SERS substrates, researchers can select the most suitable platform for their DNA sensing applications, advancing the development of sensitive and reliable diagnostic technologies. Generally, the SERS substrates used in this field are metallic nanoparticle/nanostructure-based [9,17,25,26,43,48,49,50,51,52,53,54,55,56,57,58,59,60,61]. These substrates usually require the attachment of DNA probes to the nanoparticle surface to allow the specific capture and detection of target DNA sequences. Metal nanoparticle substrates provide significant enhancement and consistent performance, making them ideal for precise DNA detection. The importance of substrate selection and the advantages/disadvantages of the various fabrication approaches will be discussed in Section 2.

The primary parameter used to characterize SERS substrates is the enhancement factor (EF). Due to the complexity of the SERS phenomenon, the large variety of substrate types, and the diverse application areas, many approaches exist to quantify the SERS EF, like the single-molecule EF (SMEF), analytical EF (AEF), and SERS substrate EF (SSEF) [62]. Generally, all of these characterize the enhancement in Raman signal intensity obtained from SERS compared with normal Raman scattering but considering different conditions and analyte concentrations. Numerous investigations focused on collecting and comparing these different EFs [63,64,65,66,67]. Studying the enhancement factor assists in comprehending how well the substrate may increase Raman signals and its ability to identify specific analytes like DNA; however, the direct comparison of the performance of different SERS substrates is remarkably impaired using different EF definitions. For a given SERS substrate, there could be orders of magnitude of differences in the EF values determined according to different EF definitions. As demonstrated by Le Ru et. al, there can be a 10^4^–10^6^ factor difference between the measured AEF and the SMEF measured with the same substrate and under the same experimental conditions (rhodamine 6G measured with colloidal aggregates) [62]. Thus, the published EF values should be treated with due caution.

DNA probe immobilization and target DNA hybridization are crucial aspects that impact sensitivity, specificity, and reliability. Immobilizing DNA probes entails binding complementary oligonucleotide sequences to the SERS substrate surface, facilitating specific capture of target DNA molecules via hybridization. Effective immobilization maximizes DNA probe surface coverage and reduces non-specific binding, improving the accuracy of DNA detection. Depending on the substrate material and test needs, immobilization procedures can be chosen, including self-assembly, covalent bonding, or biotin–streptavidin interactions. Ensuring the substrate surface is adequately functionalized with DNA probes is essential for accurate and precise DNA detection [68].

Buffer selection also plays a critical role in SERS-based DNA detection as it impacts the stability of the DNA probes, the effectiveness of hybridization, and the reliability of SERS measurements. When choosing a buffer for SERS-based DNA detection, factors include pH, ionic strength, salt concentration, buffer components, stabilizing agents, and optical transparency [68,69,70].

In the next sections, our review will first focus on the different approaches to fabricate SERS substrates tailored for DNA detection, and then the most important factors and conditions that affect the measurements will be discussed, based on the surveyed examples. Finally, our recommendations for designing SERS experiments for DNA detection will be discussed in detail. For more relevant information on this topic, we recommend the following reviews: [71,72].

## 2. SERS Substrate Fabrication Technologies

**Figure 1:**
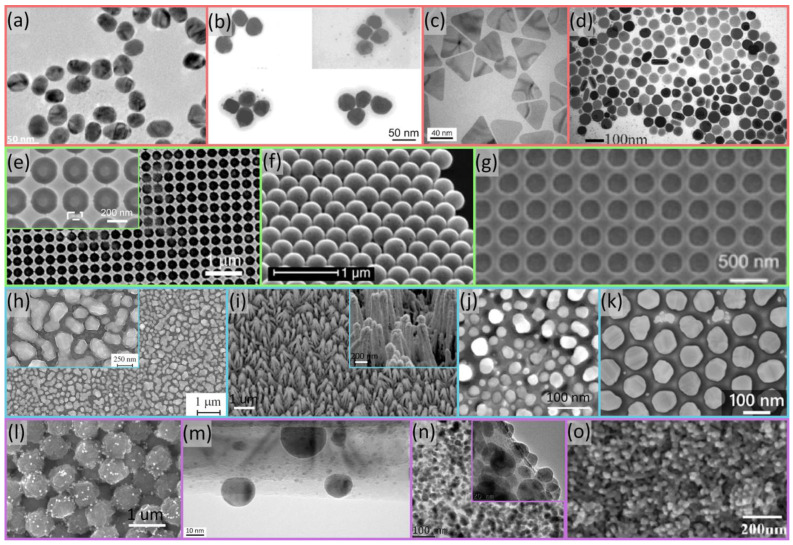
Typical SERS substrates used for DNA detection, categorized. Top row (red frame): individual nanoparticles and nanoparticle assemblies. (**a**) Spherical gold nanoparticles [73]; (**b**) silver on gold nanoshell clusters [60]; (**c**) flat silver nanoprisms [74]; (**d**) silver nanodiscs [75]. Second row (green frame): nanoparticle/nanostructure arrays. (**e**) Silicon nanostructures coated with gold–silver layer [59]; (**f**) gold nanoshell arrays [76]; (**g**) gold nanoholes [77]. Third row (blue frame): hierarchical nanostructures and semi-arrays. (**h**) Hierarchic silver clusters inside of silver rings [54]; (**i**) GaN structures coated with 70/30% gold/silver alloy [53]; (**j**) Ag nanoparticle semi-array grown into anodic aluminum oxide (AAO) template [43]; (**k**) hexagonally arranged gold nano-ellipsoids formed on the AAO template [78]. Bottom row (violet frame): heterostructures and composites. (**l**) Ultrabright nanorattles attached to magnetic microbeads [79]; (**m**) silicon nanowire (SiNW) coated with silver nanoparticles [80]; (**n**) silver nanoparticle (Ag NP)–graphene oxide (GO) nanosheet composite [81]; (**o**) gold and silver nanoparticles distributed in a graphene oxide mesh [58].

In this section, the most widespread fabrication technologies that are used to prepare SERS substrates for DNA detection are surveyed through examples from the literature. Selecting the optimal technology for a given application is not easy and often requires compromises. The most important requirements from SERS substrates include (1) high hotspot density on the surface to maximize the sensitivity and limit of detection (LOD); (2) the tunability of the plasmon absorption peak to match the wavelength of the Raman excitation; (3) the uniformity of structures on the surface to provide reproducible, quantitative data between measurement spots; (4) stability, robustness, and compatibility with the measurement environment; and (5) scalability to enable large-scale fabrication and cost-effective manufacturing. Figure 1 presents several examples of SERS substrates, which were used for DNA detection, categorized into four categories (marked with colored frames in Figure 1): individual nanoparticle or nanoparticle assembly-based substrates (red); nanoparticle/nanostructure arrays (green); hierarchical nanostructures and semi-arrays (blue); and heterostructures and composites (violet).

The plasmonic characteristics of metal nanoparticles can be tuned by adjusting their composition, size, shape, and localization (density) to enhance their effectiveness in DNA sensing tasks. First, the plasmon absorption peak of the substrate should match the Raman excitation wavelength, which typically spans from 488 nm to 785 nm. This can be best achieved by picking the right material type for the laser. Gold and silver are the two primary metals used in SERS substrate fabrication, with silver having a plasmon absorption band in the blue range, while gold has one in the green–red range, tunable to an extent to the size and density of nanoparticles [82]. Gold shows superior stability and biocompatibility; its importance for SERS applications is reviewed by [15].

SERS substrates increase the electromagnetic field confinement and amplify Raman signals in hotspots. In order to maximize the SERS signal—and gain the best possible sensitivity and LOD—the surface density of the hotspots should be maximized. This requires precise control over the size and density of the nanostructures. The size of the hotspot should ideally be comparable to that of the target molecule. Since the diameter of a ds-DNA chain is between 2 and 3 nm and its length is 0.34 nm per base pair [83], in practical cases the desired hotspot size is in the 2–10 nm range for DNA detection. 

As the measured SERS signal intensity depends severely on the number of excited hotspots, their uniformity on the surface is essential to ensure spot-by-spot reproducibility and to obtain reliable quantitative data. This is also a strong requirement for the fabrication technologies.

Nanostructured surfaces can be fabricated using processes suitable for integration into microfluidic systems, allowing them to be used in lab-on-a-chip devices for portable DNA sensing applications. All such fabricated nanostructures should be robust and stable when exposed to flowing sample conditions. 

In the next sub-sections, the most common fabrication technologies are discussed in more detail, focusing on how well they can fulfill these imposed requirements.

### 2.1. Chemical Methods

Chemical synthesis methods, such as controlled reduction and the seeded growth technique (SGT), offer precise control over the size and shape of nanoparticles. Diverse shapes of usually highly symmetric nanoparticles (spheres, rods, prisms, pyramids, core–shell structures, etc.) can be grown from a wide variety of materials. By creating anisotropic shapes (e.g., with sharp edges, tips, or high-aspect-ratio features, such as nanostars, nanotrees, or nano-sea urchins), the field enhancement around the nanoparticles can be significantly increased. The resulting nanoparticles can be used further in a colloidal form, or can be immobilized on the surface of carriers, including patterned substrates or other nanostructures as well, leading to heterogenous or hybrid nanostructures. This immobilization part is a critical step of this technology family, as controlling the density of the particles—in order to maximize hotspot density—without or with controlled particle aggregation is hard, especially when carrying this out so that the particles are in a uniform manner. Another disadvantage of wet chemical synthesis is that it is increasingly hard to synthesize complex 3D-shaped metallic nanoparticles of low symmetry as they are not favored energetically [84].

Many researchers use colloidal suspensions of nanoparticles for SERS enhancement due to the easy preparation and tunability of the plasmon resonance peak. Tunability is generally achieved by changing the colloidal structures’ size, shape, type, or composition. Synthesis of nanoparticles using this method was first described by Turkevich and Frens [85] as they fabricated gold colloids via chemical reduction. Their process was considered simple and fast and produced highly active SERS substrates. They noticed the enhanced SERS signals of nucleobases by using an aggregation agent (MgSO_4_). With this method, it is essential to use a protective agent to avoid nanoparticle aggregation, which results in better sensitivity and stability. The EF depends on the morphology of the particles, optimizing the distribution of the locally enhanced electromagnetic field above the aggregates and the adsorbed molecules.

V. Kattumuri, et al. [86] fabricated agarose-stabilized gold nanoparticles (AuNPs) to detect micromolar concentrations of DNA nucleotides. They mentioned that agarose ensures the non-degradation of probe molecules, and the agarose matrix facilitates SERS activity enhanced by one order magnitude compared with that of commercially obtained citrate-stabilized gold nanoparticles. Furthermore, its properties make it a good choice for on-chip biosensing applications. They reported on their ability to control the particles’ size using this process, as increasing the concentration of the reducing agent yielded larger nanoparticles because of the sudden increase in the local concentration of the reducing agent around gold ions inside the gel matrix. They found that the optimal number of particles was 50 in a 200 × 200 nm^2^ area and that higher particle densities resulted in lower SERS enhancements, since under higher particle densities the internal plasmon interactions between particles became delocalized. They claimed that although other researchers generally try to avoid aggregation, they found it to be necessary in a controlled amount to create hotspots on the surface. 

Xiong, et al. [87] fabricated a direct, label-free, non-destructive sensor for unmodified mushroom DNA. They prepared concentrated Ag colloids using the microwave heating method [88]. They achieved colloids of uniform size by observing the narrow LSPR peak of the Ag NPs. They reached the highest EF in the case of larger Ag colloids in the range of 40–60 nm. SERS measurement was performed with the presence of R6G. Also, they observed that using a higher concentration of Ag colloids led to a higher electric field enhancement because the increased hotspot density also led to more DNA being trapped in these spots. The interaction between Ag NPs and DNA could be observed by the naked eye, as the Ag colloid changed from yellow to yellowish brown when they added the extracted DNA solution to the concentrated Ag colloid. 

SGT is one of the methods used by many groups to fabricate monodisperse nanoparticles of a controlled shape, size, diameter, and morphology to be used for different applications as it is reported to be of low cost with simple and controllable production [47,50,74,79]. Bi, et al. [47] synthesized gold nanoplates at room temperature with this method. A simple, fast, low-cost, label-free method based on large-scale nanoplate films was demonstrated to detect DNA at room temperature without extra procedures to prevent the evaporation of the solution during measurement. They reported that the EF of SERS is highly connected to the gold nanoplate density, as a higher EF is achieved with a higher density. Also, the increased density of nanoplates resulted in the redshift and broadening of the resonance peak. The edge length also affects the EF of SERS, as the report shows an increase in the EF with an increase in the edge length, originating from the increased local electromagnetic field. 

Silver nanoprisms (NPRs) have received considerable attention as their in-plane dipole plasmon absorption band can be tuned across the visible spectrum from around 400 nm to the near-infrared (NIR) range. Silver nanoprisms can be synthesized via either photochemical (plasmon-driven) [62,85,89] or thermal synthesis [55,63,64,65,66,67,90,91,92,93,94]. 

Photochemical syntheses have produced the highest-quality samples to date with excellent monodispersity, but this approach typically requires days to prepare a sample. Thermal methods are much quicker but often lead to samples with diverse shapes and sizes [74]. 

Min Liu, et al. synthesized stable oligonucleotide-modified Ag nanoprism conjugates as a direct SERS DNA detection in aqueous solution. Ag NPR conjugates were prepared using the seeded growth method within gold nanoparticles, AuNPs, which were synthesized via chemical reduction [50].

Ahearne and co-workers [74] produced silver nanoprisms selectively (>95%) at room temperature in a rapid and reproducible manner. They tuned the spectral position of the plasmon peak by varying the size of the Ag prisms through achieving a slight change in their thickness by controlling the number of seeds in the mixture. They achieved this result by adding poly(sodium styrene sulphonate) (PSSS) as a stabilizer. Without PSSS, a wide range of prism sizes and shapes are introduced. They reported that the amount of citrate might play an essential role in anisotropic growth by influencing the defect structure of the seeds. The Ag nanoprisms showed a redshift in the resonance peak with increasing edge length, and a decrease in plasmon damping associated with increasing nanoprism size was also noticed. 

In summary, the SGTs are used by many researchers, simple, easy, and effective for fabricating different SERS substrates capable of DNA detection. Although not all the articles reported the determination method [79] or the achieved SERS EF [50], they obtained EF values with SERS substrates prepared by SGT ranging from 10^7^ to 10^11^.

### 2.2. Self-Assembly

**Figure 2:**
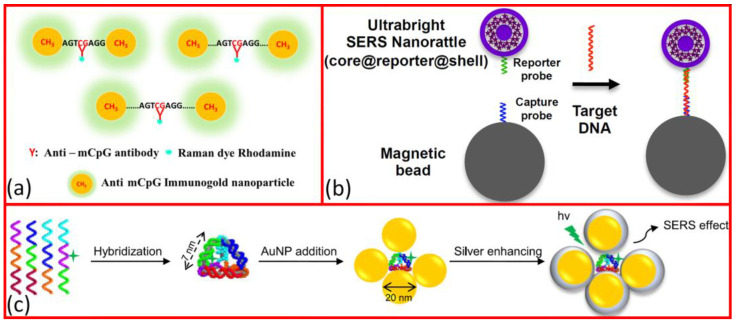
Nanoparticle assemblies used for DNA detection with SERS. (**a**) Illustration of the binding of colloidal gold spheres into dimer structures with DNA linkers of different lengths in order to tune the interparticle gap and plasmonic coupling between the particles, using rhodamine Raman labels [85]. (**b**) DNA detection scheme utilizing the assembly of ultrabright SERS nanorattles (containing HITC Raman labels) into magnetic microbeads (as shown in Figure 1l through captured and reported probe-DNA fragments [79]. (**c**) Synthetization scheme of gold–silver core–shell nanoclusters with DNA self-assembly (structure shown in Figure 1b) [60].

An interesting possibility regarding nanoparticles prepared via wet chemical synthesis is the creation of pre-designed three-dimensional clusters by using self-assembly processes. The formation of nanoparticle clusters can be controlled by either oligonucleotide-directed self-assembly [60,79] or by using templates, such as in the case of nanotrench-guided self-assembly [56]. Examples of these methods are presented in Figure 2a–c. A distinct advantage of this approach is that the hotspots between the nanoparticle structures are directly tailored to the investigated molecules, optimizing the resulting SERS signal. Furthermore, due to the reversible nature of DNA hybridization, structural/conformational changes can be imposed upon the interaction with the target DNA molecules, causing distinct changes in the optical response of the system. Keum et al. [60] created silver-enhanced gold clusters on DNA pyramids and demonstrated the on/off switching of the SERS signal through the conformational change triggered by the interaction of the DNA pyramid with the target DNAs (Figure 2c). Maruoka et al. [56] used nano-trench-guided self-assembly to fabricate a single AuNP dimer on a silicon substrate with a 100 nm diameter and only a 1 nm gap. They reported 10^11^ EF for eight-base-long targets.

Thacker et al. [95] also proposed a plasmonic coupling-based sensor, based on DNA origami-assembled AuNP dimers on a gold-coated Si wafer, with a 40 nm NP particle diameter and reproducible 3.3 ± 1 nm gaps. They attached AuNPs to folded origami structures and reported that their platform showed strong plasmonic coupling between the NPs without occupying the gap between them. They performed SERS measurements on individual dimer structures and recorded an EF of 10^4^–10^5^ for 20-base-long DNA. 

Plasmon coupling-dependent SERS was also demonstrated by Nguyen et al. [73], who designed a fast method to detect global DNA methylation with SERS using a coupled gold nanoparticle (AuNP)–silver nanowire (Ag NW) structure on glass. They used anti-mCpG immunogolds to bind to the mCpG sites on the DNA sequences, by following standard methods of chemical reduction by Turkevich and Frens [85]. They controlled the gap between AuNPs aligned on DNA by controlling the modification sites on the DNA backbone in the range of 3.4–68 nm (illustrated in Figure 2a) and kept AuNPs within the plasmon coupling distance. The highest SERS intensity was obtained at a distance of 17 nm. The LOD of global methylated DNA with their platform was 18 fg/mL^−1^, which is still lower than that obtained via PCR-based detection followed by pyrosequencing.

To overcome the challenges of achieving both ultrahigh sensitivity and good reproducibility, Li et al. proposed a strategy called the DNA-assisted synthesis of ortho-nanodimers (DaSON) from Au and Ag particles for SERS sensing. In their design, the two nanoparticles are constrained by the equilibrium state of DNA hybridization and electrostatic repulsion to form zipper-like ortho-nanostructures [96], with a tunable, uniform gap between the particles. With their design, they significantly increased the SERS signal and reached an LOD of 100 attomole for target DNA detection.

### 2.3. Physical Methods

Physical methods, namely the deposition of thin metallic films on substrates and their subsequent patterning with lithography or other processing methods, are a straightforward way to grow nanostructures directly onto solid substrates, without the need for surface chemistry of stabilizing ligands. There are two standard deposition techniques with which to obtain good-quality thin films: physical vapor deposition (PVD, such as vacuum sputtering or evaporation) and versatile chemical vapor deposition (CVD) [97]. Specific fabrication methods may differ in their approach, whether they create a homogenous thin film on a flat substrate first, which they subsequently pattern with lithography, or they use a nano-structured template to coat with thin film, prior to subsequent processing steps. In this section, we will discuss some examples of research that followed this method to fabricate SERS substrates used for DNA sensing purposes.

Fang et al. [59] used standard silicon process technologies, such as deep UV photolithography, to fabricate silicon nanostructures, which they subsequently coated via physical vapor deposition, using silver as a bottom layer and gold for the top layers. With the resulting nanogaps of a 15 ± 10 nm distance, they were able to detect target DNA by using peptide nucleic acid (PNA) probes and rhodamine 6G indicator molecules [59].

Frøhling et al. [61] used maskless reactive ion etching (RIE) to create silicon nanopillars with a 500 nm height and 100 nm diameter, which they subsequently coated with 200 nm gold via e-beam deposition to create a 2D SERS substrate. They investigated the effect of DNA concentration and incubation time on the obtainable SERS signal.

Coluccio et al. [49] used electron beam lithography to create a template for subsequent electroless silver deposition to realize a self-similar chain of nanospheres (with a diameter of 10 nm) on a layer of electronic resist spun onto a silicon wafer. They were able to detect short DNA chains (with 6–9 bases) inside the gaps between the spheres.

Physical methods can also be combined with chemical etching techniques to provide templates for subsequent deposition and patterning. Chan et al. [43] proposed an electrochemical etching process to prepare a nano-island of Ag NPs on a porous anodic aluminum oxide (AAO) template (the realized structures are shown in Figure 1j). The AAO nanochannels were chemically etched, creating a pore diameter of 20–40 nm and a pore-to-pore distance of 10–20 nm, and the Ag nanoparticles were grown in the AAO nanochannels via electrochemical plating. They measured the SERS spectra of adenine (10^−4^ M) at seven different spots separated by about 10 to 15 mm on the AgNP/AAO substrate and obtained an EF > 10^8^.

**Figure 3:**
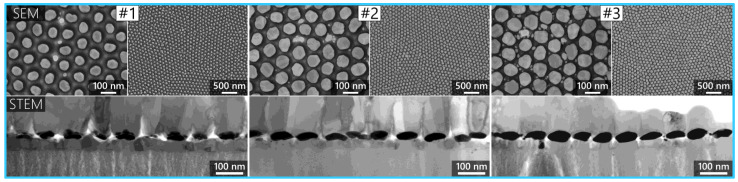
Top row: SEM images of three different AuNP arrangements over a porous aluminum template, created via solid-state dewetting. Bottom row: STEM (BF) images of the same AuNP arrangements of SERS substrates on the SiO_2_ nanopillars/substrate. The three numbered stages demonstrate the tunability of the particle arrangement. Reproduced from [78].

Lednický and Bonyár also used AAO-based templates combined with solid-state dewetting to create hexagonally arranged, ellipsoidal gold nanoparticle semi-arrays on a large surface area (cm^2^ range, the structures are shown in Figure 1k). After synthesis, they transferred the nanoparticle arrangement onto polymer or glass substrates and used the nanoparticles as LSPR sensors and SERS-substrates [82,98]. They managed to tune the particle size (see Figure 3) and showed that by decreasing the interparticle gap between the particles, the EF increases upon DNA detection [82].

### 2.4. Hybrid Nanostructures

By combining the above-mentioned technologies (chemical and physical methods), we can prepare hybrid nanostructures and heterostructures that combine the advantages of different materials, structures, and technologies to increase SERS substrate performance even further. The combination of nanostructures with different size ranges can help in the design of hotspot size and in the control of particle aggregation (e.g., the decoration of nanowires with smaller nanoparticles). The combination of different materials can be beneficial to utilize different SERS enhancement approaches (chemical and electromagnetic) together.

Ngo. et al. (2017) proposed a simple and easy method for DNA detection with single-nucleotide polymorphism (SNP) that is suitable for integrating into lab-on-a-chip systems for POC molecular diagnostics by utilizing gold nanoparticles (AuNPs) in a detection scheme with sandwich hybridization [79]. It consists of an ultrabright SERS nanorattle (see Figure 1l), which has a core–gap–shell structure with Raman reporters loaded in the nano-sized gap between the core and the shell, as shown in Figure 2b. They reported that these structures have significantly higher enhancement than simple gold nanospheres using the same reporters. The SERS activity of core–shell NPs is affected by the size of the core and the type and thickness of the metallic shell [76,99,100].

Kamińska et al. [53] synthesized a novel Au–Ag coated GaN SERS nanocomposite by combining many techniques (metal–organic chemical vapor deposition (MOCVD), photo etching, and sputtering) for Au and Ag deposition. GaN epitaxial layers grown on sapphire via MOCVD were subjected to photo-etching in alkaline solutions, revealing dislocations and electrically active defects in GaN and SiC [101], which were analyzed, creating pillars around these linear defects, and after long etching, forming “sheaves”. A metallic layer (gold with silver in 30–70%) was deposited on photo-etched GaN samples via a sputtering technique. The resulting structures are shown in Figure 1i. The SERS study showed the excellent reproducibility and stability of the surface.

Via a labeled method, Gaidi et al. [80] used pulsed laser deposition to fabricate Si-NWs decorated with Ag NPs. The structures are shown in Figure 1m. They used rhodamine 6G (R6G) as a label to detect the target DNA. 

**Figure 4:**
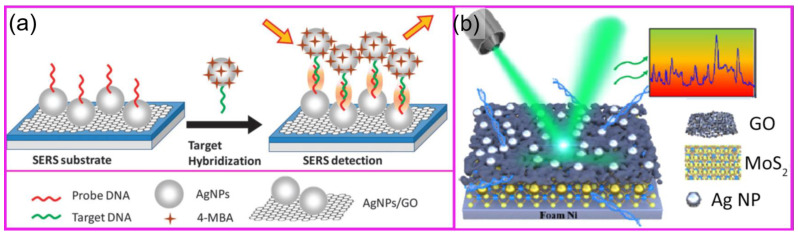
Strategies to fabricate hybrid nanostructures for SERS. (**a**) Target DNA detection scheme with an Ag NP–Ag NP–GO heterostructure, using 4-MBA Raman label molecules. The structure is presented in Figure 1n [81]. (**b**) Label-free detection of DNA with an AuNP @ GO mesh @ Ag NP heterostructure. The structure is presented in Figure 1 [58].

Graphene and graphene oxide (GO) are considered promising SERS substrates for DNA detection because of their distinctive electrical and optical characteristics. Graphene’s unique properties, such as fluorescence quenching and chemical enhancement, can increase the SERS signal of molecules adsorbed on its surface. Although chemical enhancement is usually orders of magnitude weaker than EM enhancement, the combination of graphene with plasmonic materials such as Au or Ag NPs can enable the utilization of both approaches at once [74]. Furthermore, graphene-based substrates provide enormous surface areas, superior biocompatibility, and chemical stability, making them ideal for anchoring DNA probes and trapping specific DNA molecules. Novel hybrid nanostructures, incorporating metal nanoparticles, graphene, and semiconductor nanomaterials, have been created to boost SERS signals for DNA detection collectively. These hybrid substrates utilize the unique characteristics of each material component, like plasmonic enhancement, charge transfer, and surface functionalization, to boost DNA detection performance. Figure 4 presents two technological approaches to preparing SERS substrates combined with GO.

## 3. Conditions Affecting SERS Measurements

As has been discussed above, SERS relies on enhancing the Raman signal by several orders of magnitude through the nanostructure’s localized surface plasmon resonance. Several conditions affect the efficiency of the SERS effect: the laser wavelength and power, and the SERS excitation conditions.

SERS enhancement requires the wavelength of Raman excitation (λ_exc_) and scattering (λ_RS_) to match the plasmonic resonance absorption peak (λ_SP_). Theoretical and experimental results have proven that the maximum enhancement occurs when λ_SP_ is at the midpoint between λ_exc_ and λ_RS_, that is, when λ_SP_ = 0.5(λ_exc_ + λ_RS_) [88,101,102,103,104,105]. While λ_exc_ is determined by the Raman spectroscopic system used for the measurements, λ_RS_ depends on which Raman bands are to be investigated. Most of the DNA SERS studies focus on the fingerprint region between 1000 and 1800 cm^−1^, but in some cases, C-H stretching in the 2700–3100 cm^−1^ region can also be targeted. The difference between the two can be 1000–2000 cm^−1^, which (depending on the excitation wavelength used) represents a 70–150 nm difference compared with the optimal λ_SP_. 

As Table 1 shows, SERS measurements of DNA were performed in a broad range of Raman excitation wavelengths, from the visible to near-infrared region, mainly covering the most widely used laser sources: 532, 632, and 785 nm. While a shorter wavelength will excite a stronger Raman signal (the Raman intensity is proportional to λ_exc_^−4^), it could also be a source of strong photoluminescence that can overlap the Raman bands. Therefore, in general, the use of near-infrared Raman excitation is preferred for biological samples; however, this is not true for the non-luminescing DNA, except if there are contaminants in the sample with strong emission. 

The laser powers used for the experiments vary between 12 μW and 300 mW (see Table 1). Although the values spread by three orders of magnitude, this is mainly caused by the fact that some papers provide the power of the laser integrated into the Raman system, while others give the laser power value measured at the sample. In addition, by changing the focusing conditions, the same laser power could excite Raman scattering at different laser intensities; therefore, in addition to the wavelength, the latter is the objective marker of the Raman excitation conditions. The higher the laser intensity, the better the Raman signal; however, high intensities could cause alteration, heating, and even destruction of the sample. In the case of Ag nanoparticles, photo-oxidation caused by near-UV light that could lead to a degradation in SERS performance was also reported [106]. 

The plasmon resonance wavelength of the SERS substrate depends on the properties of the surrounding medium, which could be affected by the analyte itself. For example, a roughly 4 nm redshift of the λ_SP_ of Ag NPs was observed after the addition of the DNA solution to an Ag colloid [87]. This must be considered when selecting the SERS substrate for DNA measurement; however, this change can also be used as an indicator of the successful binding of the DNA to the SERS substrate.

In addition to the above, the properties of the surrounding medium also affect the binding of the analyte to the SERS substrate, being crucial for maximizing Raman signal enhancement. Typically, molecules interact with the metallic surface through weak long-range forces. In the case of colloidal solutions, temperature, pH, and ionic strength can influence the adsorption kinetics. 

Numerous research groups have investigated the impact of pH on the SERS intensity of organic molecules [107,108,109,110,111,112,113,114]. It was shown with adenine as the example that the change in the pH (between 2.5 and 11.5) affects the orientation of the nucleobases relative to the SERS substrate, resulting in changes in the Raman peak intensities and the shifting of some of the Raman bands [115]. In addition, reduced binding to the gold surface was observed at low pH values. These findings highlight the importance of performing (comparative) DNA SERS experiments under the same conditions.

The proper ionic strength of the DNA solution is also essential for achieving optimal efficiency in hybridization [116]. A study involving organic dyes dispersed in aqueous solutions spanning a pH range of 3 to 11 revealed that, in addition to the different affinities of the dyes for silver and gold, SERS activity was also influenced by the pH. Remarkable differences were observed between normal conditions (pH 7) and pH values of 3 and 11, depending on both the dye and the type of metal involved [109]. In this regard, the use of deionized water for rinsing substrates covered with DNA could also influence the SERS experiment [47,48,55,96]; after hybridization, this could expose the DNA strands to possible denaturation by abruptly dropping the ionic strength [117]. 

To achieve efficient SERS measurements, aggregation agents can be utilized to improve the properties of the surrounding medium. For example, 0.01 M MgSO_4_ was used to neutralize the surface charge of Ag nanoparticles and introduce more hotspots through their aggregation [55]. The DNA was found to be trapped in these hotspots, resulting in an extremely increased DNA SERS signal. On the other hand, a dramatic decrease in the DNA SERS signal was observed when a high concentration of NaCl was used as an aggregation agent [48], which was explained by the intense competition between the chloride anions and DNA. 

The size of the investigated target molecules should also be considered with respect to the size of the designed hotspots. As can be seen in Table 2, the size of the targets varied a lot, from single nucleobases and shorter ss-DNA chains to longer ds-DNA oligomers. Smaller molecules fit easily into hotspots; however, depending on the design of the substrate, it might be harder to incorporate longer targets into the most sensitive regions between the nanoparticles. Besides spatial hindrance, too much aggregation of the nanoparticles may cause them to lose their plasmonic properties at the desired wavelength. Increasing the coupling between the particles by decreasing the interparticle gap shifts their plasmon resonance peak towards longer wavelengths (which should also be considered during substrate design) [118]; touching particles (larger aggregates) may lose their plasmonic properties entirely. 

Conclusively, we could say that the interparticle distance has a theoretical optimum for every target molecule/experimental condition (e.g., excitation wavelength) pair, where the field strength is maximal between closely packed particles—at a controlled plasmon resonance wavelength—but the size of the hotspots still provides accessibility for target molecules With this optimum particle density, the SERS response is maximal, while both below and above this, it is expected to decrease, as demonstrated and discussed in [86].

## 4. Designing SERS Experiments for DNA Detection

In general, three approaches can be used to design SERS experiments: those driven by the DNA to be detected, by the Raman instrumentation, and/or by the SERS substrate. In an ideal case, the conditions of the SERS measurement are determined by the scientific question to be answered, i.e., the type, form, concentration, cleanliness, etc., of the DNA sample to be detected. Then, the best SERS substrate can be selected based on these parameters. In some cases, the desired SERS substrate is not available or complicated to prepare. This means that an alternative must be synthesized or purchased, with parameters close to the original one. In the last approach, the Raman instrumentation was given, including the available excitation sources. Then, the main constrain was the excitation wavelength that determined which SERS substrates could be used for the measurements, in terms of the material, and the nanoparticle/nanostructure size and shape. 

It can be seen in Table 2 that gold, silver, and gold–silver SERS substrates were demonstrated to be suitable for DNA detection. Most of the studies were performed with metallic nanospheres prepared via wet chemical synthesis. Amongst the 18 reports evaluated, seven used Au + Ag, six Au, and five Ag particles as the material of the SERS substrate. As expected, the selection of the metal correlates with the Raman excitation wavelength: silver was used with shorter wavelengths (488–632 nm), and gold was used with longer wavelengths (632–785 nm), while the gold–silver substrates covered a broad range of Raman excitations (514–785 nm).

The DNA concentrations investigated were between 10^−4^–10^−15^ M and 10^−2^–10^−11^ mg/mL. The lowest values represent the limits of detection achieved by SERS substrates of Si nanowires decorated by Ag NPs and combined silver nanowires and gold nanoparticles. It can be seen in Table 2 that the detection of DNA in 10^−12^–10^−15^ M concentrations with SERS was achieved by several groups. The best LOD (10^−15^ M) was demonstrated with DNA origami structures, using gold nanoparticle dimers with a 5 nm gap, filled with the DNA helices [80]. The detection of femtogram per milliliter concentrations of methylated DNA was shown with a SERS substrate consisting of coupled gold nanoparticles–silver nanowires [73].

Despite the approach used, the main task is to match the excitation source, the SERS substrate parameters, and the DNA detection method, taking into account the considerations described earlier. By knowing the Raman spectral region of interest (λ_RS_) and the excitation wavelength (λ_exc_), the LSPR wavelength of the SERS substrate (λ_SP_) can be determined based on the λ_SP_ = 0.5 (λ_exc_ + λ_RS_) rule. Then, based on the detection method (label-free or label-based) and the targeted DNA concentrations, a suitable SERS substrate can be selected, together with the proper sample preparation method.

**Table 2:** Technological and instrumentational parameters of the surveyed Raman investigations for DNA detection. Acronyms: 4-MBA: 4-mercaptobenzoic acid; AAO: anodic aluminum oxide; Ag IANP: (I^−^ and Al^3+^) modified silver nanoparticle; Cy3: (1,1′-bis(3-hydroxypropyl)-3,3,3′, 3′-tetramethylindocarbocyanine); dA: 2′-deoxyadenosine; DCM: dichloromethane; dCMP: 2′-deoxycytidine-5′-monophosphoric acid; dGMP: 2′-deoxyguanosine-5′-monophosphate; ds-DNA: double-stranded DNA; dT: 2′-deoxythymidine; DTNB: 5,5′-dithiobis (2-nitrobenzoic acid); GNP: gold nanoplate; GO: graphene-oxide; HITC: 1,3,3,1′,3′,3′,-hexamethyl-2,2′-indotricarbocyanine iodide; MGITC: green malachite isothiocyanate; MOCVD: metalorganic chemical vapor deposition; PNA: peptide nucleic acid; RB: Rhodamine B; R6G: rhodamine 6G; ss-DNA: single-stranded DNA; TP: thiophenol.

Ref	Material Type	Shape/ Structure	Size/ Geometry	Fabrication Technology	DNA Concentration Range/Value	Limit of Detection (LOD)	Target Type, Length, Raman Label	Enhancement Factor (EF)	SERS Parameters: Wavelength (*λ*), Power (*p*), and Acquisition Time (*t*)
[47]	Au	nanoplate	edge length = 134 ± 6 nm, density = 916 ± 40 GNPs/spot	Wet chemical synthesis (seed-mediated growth) of nanoplates + self-assembly on glass	10^−2^–10^−6^ mg/mL	10^−6^ mg/mL	nucleobases (A,T,G,C) label-free	5.4 × 10^7^	*λ* = 785 nm, *p* = 9.5 mW
[50]	Au + Ag	nanoprism/ nanosphere	Ag nanoprism edge length = 110 ± 10 nm Au nanosphere dimeter = 25 ± 5 nm	Wet chemical synthesis (seed-mediated growth) of Ag nanoprism + hybridization with AuNP-modified DNA	10^−8^–10^−11^ M	10^−11^ M	ss-DNA 30 bases Raman labels: DTNB and 4-MBA	-	*λ* = 632.8 nm, *p* = 2.3 mW
[53]	Au + Ag coated GaN	nanopillars	thickness = 90 nm	GaN epitaxial growth (MOCVD) + photo-etching + physical deposition (Au + Ag) + dealloying	10^−5^ M	-	ss-DNA 22 bases Raman label: MGITC	1 × 10^7^	*λ* = 632.8 nm, *p* = 5 mW, *t* = 10–30 s
[56]	Au	nanosphere dimers	gap = 1 nm, dimeter = 100 nm	Nanotrench-guided self-assembly on patterned Si	-	10^−11^ M	ss-DNA 8 bases label-free	10^11^	*λ* = 632.8 nm, *p* = 8.2 mW, *t* = 0.5 s
[49]	Ag	nanospheres (self-similar chains)	*d*_1_ = 148 ± 30 nm, *d*_2_ = 64 ± 6 nm, *d*_3_ = 27 ± 5 nm, gap_23_ = 10 nm, gap_12_ = 31 nm	E-beam lithography on Si + electroless Ag deposition	10^−8^ M	-	ss-DNA 6–9 bases label-free	10^12^	*λ* = 514 nm, *p* = 0.012 mW
[57]	Au	nanospheres	dimeter = 60 nm, gap ≅ 5 nm	Wet chemical synthesis + DNA-controlled aggregation	-	10^−7^ M	ss-DNA (12 bases) ds-DNA (24 bases) label-free	2.2 × 10^5^	*λ* = 632.8 nm, *p* = 10 mW
[58]	Au + GO + Ag	AuNPs@ GO mesh@ AgNPs	AuNP diameter = 40 nm; AgNP diameter = 50 nm	Chemical reduction of hAuCl_4_ on GO/MoS_2_ + chemical synthesis and deposition of Ag NPs	-	10^−13^ M	ss-DNA (9 bases) ds-DNA (12 bases) label-free	4.2 × 10^8^	*λ* = 532 nm, *p* = 0.5 mW
[59]	Au + Ag on Si	Nanogaps	gaps = 15 ± 10 nm, Ag thickness = 30 nm, Au thickness = 15 nm	Photolithography + reactive ion etching for the Si substrate + physical deposition (Au, Ag)	10^−8^–10^−12^ M	10^−12^ M (using RB label)	PNA-DNA complex (22 bases) Raman label: RB	-	*λ* = 785 nm, *p* = 300 mW
[43]	Ag	Ag NPs on AAO substrate	Ag NP diameter = 20–50 nm, AAO pore size = 20–40 nm, gap = 10–20 nm	Anodic oxidation and wet chemical etching (AAO) + Ag electrochemical plating	10^4^–10^−4^ ppm	10^−3^ ppm	nucleobases (T,G,C) label-free	1.9 × 10^8^	*λ* = 632.8 nm, *p* = - *t* = 60 s
[60]	Au + Ag	Au@Ag core–shell structures	Au nanosphere diameter = 20 nm Au@Ag core–shell diameter = 35 nm	Wet chemical synthesis + DNA self-assembly into nanopyramids	10^−9^ M	-	ds-DNA (DNA-pyramid) Raman label: Cy3	-	*λ* = 514.5 nm, *p* = 20 mW, *t* = 10 s
[61]	Au on Si	coated nanopillar	Si nanopillar height and diameter: 500 nm × 100 nm Au layer thickness: 200 nm (2D arrays 4 mm × 4 mm)	Reactive ion etching of Si (randomly distributed nanopillars) + physical deposition (Au)	5 × 10^−6^ M	-	ss-DNA, 75 bases, label-free	-	*λ* = 780 nm, *p* = 0.1 mW, *t* = 1 s
[48]	Ag	iodide-modified Ag nanospheres	diameter = 50 nm	Wet chemical synthesis + iodide-modification	3.5 × 10^−6^ M	-	ss-DNA and ds-DNA (10–51 bases) label-free	-	*λ* = 532 nm, *p* = 5 mW, *t* = 10 s
[86]	Au	nanospheres	diameter = 15 nm	Wet chemical synthesis (agarose-stabilized nanoparticles)	10^−4^ M	-	nucleosides (dA, dT, dCMP, and dGMP) label-free	-	*λ* = 785 nm, *p* = 5 mW
[39]	Au	Au nanoshell on glass sphere	core diameter = 120 nm	Stöber method for silica nanoparticles, wet chemical synthesis (AuNPs) + surface chemistry to create nanoshells	4 × 10^−5^ M	-	ss-DNA and ds-DNA (20–70 bases) label-free	-	*λ* = 785 nm, *p* = 0.57 mW, *t* = 20 s
[79]	Au + Ag	nanorattles (Au–Ag porous cages)	diameter ≅ 60 nm	Wet chemical synthesis (seed-mediated growth, galvanic replacement)	10^−9^–10^−13^ M	3 × 10^−12^ M	ss-DNA and ds-DNA (25–80 bases) Raman label: HITC	-	*λ* = 785 nm, *p* = 300 mW, *t* = 1 s
[81]	Ag	Ag NP + GO nano- composite	Ag NP diameter = 57.5 nm	Wet chemical synthesis (Ag NP), modified Hummers’ method (GO nanosheets)	10^−6^–10^−12^ M	10^−12^ M	ds-DNA, 20 bases, Raman label: 4-MBA, TP	-	*λ* = 532 nm, *p* = 1.5 mW, *t* = 3 s
[80]	Ag + Si	Si NW + Ag NP	Si NW length = 2.4 μm, diameter = 20–60 nm, Ag NP diameter = 40 nm	Metal-assisted chemical etching (Si NWs), Ag thermal evaporation, and pulsed laser ablation for Ag NPs	10^−10^–10^−15^ M	10^−15^ M	ss-DNA 25 bases label-free	~10^6^	*λ* = 488 nm, *p* = 100 μW, *t* = 10 s
[73]	Au + Ag	Ag NW + AuNP	Ag NW length = 24 μm, diameter = 121 nm, Au NP diameter = ~35 nm	Wet chemical synthesis for both Ag NWs and AuNPs	10^−9^ mg/mL – 1.8 × 10^−11^ mg/mL	1.8 × 10^−11^ mg/mL	ds-DNA (long oligomer) Raman label: R6G	-	*λ* = 785 nm, *p* = 8.5 mW *t* = 0.3 s
[119]	Ag	Ag DIANPs	Ag NP diameter ≅ 100 nm	Chemical synthesis: DCM-modified Ag IANPs (DIANPs)	1 × 10^−6^ M	-	ss-DNA and ds-DNA (10–50 bases) label-free	-	*λ* = 633 nm, *p* = - *t* = 10 s
[32]	Ag	AgZNPs	Ag NP diameter ≅ 36 nm	Chemical synthesis: Zr ion-modified Ag NPs (AgZNPs)	1 × 10^−5^ M	-	ss-DNA (12–35 bases) label-free	-	*λ* = 633 nm, *p* = 20 mW *t* = 30 s

## 5. Conclusions

In this review paper, the main aspects of SERS-based sensors for DNA detection were summarized, including the principles of SERS, the fabrication methods of suitable SERS substrates, conditions affecting DNA sensing, and steps of the design of a SERS-based DNA detection experiment. Detailed experimental conditions and detection limits of previous studies that can be of help in selecting and optimizing the most suitable SERS substrate and measurement methodology for nucleotide sensing applications with Raman spectroscopy were provided.

Various fabrication methods for creating SERS-based DNA sensors were discussed in detail, highlighting the advantages and disadvantages of the different approaches. By analyzing the most important conditions that affect SERS measurements and by evaluating the previously published works along these criteria, we suggested general recommendations for designing SERS experiments aimed at DNA detection.

## Figures and Tables

**Table 1:** Specific DNA-related Raman peaks and their assignments [33,34,35,36,37,38,39,40,41,42,43,44,45,46,47,48,49].

Peak Position (cm^−1^)	Assignment
471	T (ring stretching)
574	A (ring deformation)
649, 659, 656	G (ring breathing)
667–669, 675	G (ring breathing)
685	A (stretching), G (deformation (C-H))
707, 719	A (scissoring, C–S)
723–731	A (ring breathing)
745–754	T (stretching in C5–CH_3_)
750–758	T (ring breathing)
761	A (ring breathing)
783–787	C (ring breathing)
865	G (ring stretching)
828–835	Phosphodiester O–P–O stretching, T (C4–C5 stretching)
875	Deoxyribose ring
934, 936, 938, 939	A/C/G (deoxyribose stretching)
1005–1008	C–O stretch in the deoxyribose
1021–1034	T (C–N–C bending)
1061	A (C–N stretching)
1073–1080	PO_2_ stretch in backbone
1077	PO_2_ stretch in backbone
1124	A (stretching of the deoxyribose phosphate backbone)
1160	A/G/T (stretching of the deoxyribose phosphate backbone)
1190–1198	C (C–N stretching, N-H bending)
1203, 1207, 1208	T (stretching of the deoxyribose phosphate backbone)
1262	A/T (C–C and C–N stretching)
1269–1276	C (ring stretching, C–N stretching)
1290, 1299	C (CH2 deformation)
1333	A/G (CH2 wagging mode)
1341	G (C–N stretching)
1355, 1360, 1370	A/C/G/T (C–N stretching)
1398	T (NH deformation/CH_3_ deformation)
1461	A (C–H deformation of deoxyribose)
1507, 1514, 1518	C (C–N stretching, NH_2_ deformation)
1580–1590	C/G/T (C–N stretching)
1602	A/G (C=C stretching)
1643	C/G/T (C=O stretching, C=C stretching)
1670	A (NH_2_ scissoring)
1700	T (C=O stretching)

Abbreviations: A, adenine; C, cytosine; G, guanine; T, thymine.

## Data Availability

The collected data are available from the authors upon request.
